# Exercise-Based Mechanotherapy: From Biomechanical Principles and Mechanotransduction to Precision Regenerative Rehabilitation

**DOI:** 10.3390/ijms27020694

**Published:** 2026-01-09

**Authors:** Guang-Zhen Jin

**Affiliations:** Institute of Tissue Regeneration Engineering, Dankook University, Cheonan 31116, Republic of Korea; gzhjin2002@dankook.ac.kr; Tel.: +82-41-550-3082

**Keywords:** exercise-based mechanotherapy, mechanotransduction, musculoskeletal tissues, precision rehabilitation

## Abstract

Mechanical loading generated during physical activity and exercise is a fundamental determinant of musculoskeletal development, adaptation, and regeneration. Exercise-based mechanotherapy, encompassing structured movement, resistance training, stretching, and device-assisted loading, has evolved from empirical rehabilitation toward mechanism-driven and precision-oriented therapeutic strategies. At the macroscopic level, biomechanical principles governing load distribution, stress–strain relationships, and tissue-specific adaptation provide the physiological basis for exercise-induced tissue remodeling. At the molecular level, mechanical cues are transduced into biochemical signals through conserved mechanotransduction pathways, including integrin–FAK–RhoA/ROCK signaling, mechanosensitive ion channels such as Piezo, YAP/TAZ-mediated transcriptional regulation, and cytoskeleton–nucleoskeleton coupling. These mechanisms orchestrate extracellular matrix (ECM) remodeling, cellular metabolism, and regenerative responses across bone, cartilage, muscle, and tendon. Recent advances in mechanotherapy leverage these biological insights to promote musculoskeletal tissue repair and regeneration, while emerging engineering innovations, including mechanoresponsive biomaterials, 4D-printed dynamic scaffolds, and artificial intelligence-enabled wearable systems, enable mechanical loading to be quantified, programmable, and increasingly standardized for individualized application. Together, these developments position exercise-informed precision mechanotherapy as a central strategy for prescription-based regenerative rehabilitation and long-term musculoskeletal health.

## 1. Introduction

The concept of mechanotherapy dates back to the nineteenth century, originating from exercise-based Swedish Medical Gymnastics and the Zander mechanotherapeutic system. In the early 1800s, Per Henrik Ling established the Royal Central Institute of Gymnastics and proposed that systematic movement, stretching, and exercise could enhance joint mobility, improve circulation, and restore muscle strength, laying the foundation for modern physical therapy [1]. Later in the same century, Gustav Zander advanced this paradigm by developing mechanical devices that delivered traction, assistance, resistance, and massage, enabling standardized mechanical loading in clinical rehabilitation [2,3]. By the late nineteenth and early twentieth centuries, exercise-based stretching, massage, and device-assisted resistance training were widely adopted to improve soft tissue flexibility, promote blood flow, and enhance muscular strength, becoming integral components of exercise-oriented physical therapy and physical agent modalities [4].

With advances in mechanobiology and cellular mechanotransduction, mechanotherapy has evolved from empirical practice toward mechanism-based and precision-oriented strategies [5,6]. Modern mechanotherapy is defined as the application of controlled mechanical loads or stimuli to promote tissue repair, remodeling, and functional restoration. Its core principle is to harness the body’s intrinsic capacity to sense and transduce mechanical cues, thereby modulating cellular behavior and driving tissue regeneration [7,8].

Compared with conventional rehabilitation approaches that primarily target symptomatic or functional improvement, such as pain reduction, muscle strengthening, or enhanced joint mobility, mechanotherapy is grounded in mechanosensation, mechanotransduction, and mechanoadaptation. These concepts provide a biological framework for targeted, mechanism-informed interventions [5,8,9]. By directly modulating the tissue microenvironment and cellular responses, mechanotherapy not only enhances functional outcomes but also facilitates structural repair, showing strong synergy with regenerative strategies, including stem cell therapies and tissue engineering approaches [6,7].

Recent advances in mechanoresponsive biomaterials, shape-morphing four-dimensional (4D) scaffolds, and AI-driven wearable systems capable of delivering adaptive mechanical stimulation have further expanded the potential of mechanotherapy for precision and personalized interventions. These technologies enable real-time monitoring and regulation of mechanical parameters, including load magnitude, frequency, and duration, creating new opportunities to enhance therapeutic efficacy and broaden clinical applicability in musculoskeletal rehabilitation [5,6,8,10].

## 2. Macroscopic Biomechanical Principles of the Musculoskeletal System

The musculoskeletal system, composed of bones, joints, muscles, tendons, and ligaments, serves as the primary mechanical framework for supporting body posture and enabling movement [11]. Muscles generate contractile forces under neural control, transmitting tension through tendons to produce joint rotation and locomotion. Bones provide rigid levers, joints allow controlled mobility, and tendons and ligaments ensure efficient force transmission and joint stability [11,12]. Lever mechanics underlie joint movement, translating muscular effort into displacement and enabling efficient force application [11]. Furthermore, bones continuously remodel in response to mechanical loading, sustaining tissue homeostasis and functional capacity [12]. Collectively, these macroscopic biomechanical properties establish the mechanical environment that informs tissue-level mechanotransduction, providing a foundational basis for mechanotherapy.

At the organ level, the musculoskeletal system is continuously subjected to both internal and external mechanical stimuli, which not only drive movement but also regulate tissue structure and function. Key mechanical forces, including tensile, compressive, shear, and hydrostatic stress, each contribute to specific tissue adaptations that are directly relevant to mechanotherapy [13]. Tensile stress predominantly affects muscles, tendons, and ligaments by guiding fiber alignment, collagen remodeling, and tissue elasticity. In mechanotherapeutic practice, controlled tensile loading is applied through structured stretching and resistance exercises to restore flexibility, enhance muscular strength, and promote functional recovery [14]. Compressive stress primarily acts on bone and cartilage, stimulating bone remodeling and extracellular matrix synthesis. Mechanotherapy harnesses controlled compressive loading to maintain bone density, enhance cartilage resilience, and preserve joint integrity during rehabilitation [15]. Shear stress, which commonly occurs in synovial joints and the bone canalicular network, regulates cellular morphology, alignment, and secretion patterns. Therapeutic interventions that replicate physiological shear stress can optimize cartilage lubrication, support joint homeostasis, and facilitate adaptive tissue remodeling [16]. Similarly, hydrostatic pressure, prevalent in joint cavities and cartilage, contributes to the maintenance of cell volume, structural stability, and metabolic activity. Mechanotherapy employing cyclic hydrostatic loading has been shown to enhance cartilage metabolism and improve tissue repair outcomes [17].

**Figure 1:**
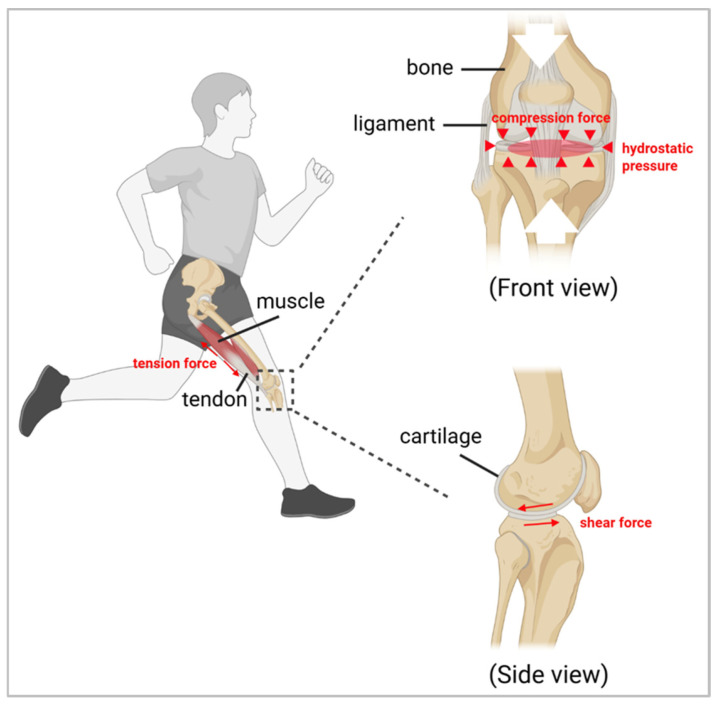
Common types of mechanical stress, including tension, compression, shear, and hydrostatic pressure, synergistically shape a dynamic mechanical microenvironment within the musculoskeletal system. This microenvironment regulates movement adaptation, injury repair, and mechanical homeostasis. Figure created with www.BioRender.com (licensed).

Together, these mechanical cues establish a dynamic microenvironment that governs tissue adaptation, injury repair, and homeostatic balance (Figure 1). By precisely applying these forces, mechanotherapy can modulate the tissue microenvironment, influence cellular responses, and accelerate functional restoration, thereby connecting macroscopic biomechanics to cellular-level mechanotransduction and targeted rehabilitation strategies [18,19,20].

## 3. Molecular Basis of Mechanotransduction

**Figure 2:**
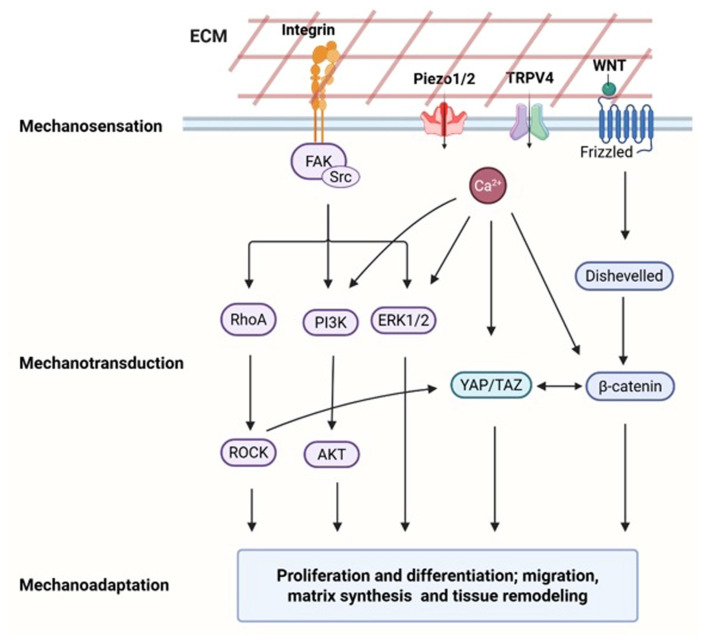
Schematic illustrating the three sequential stages of mechanotherapy: mechanosensation, mechanotransduction, and mechanoadaptation, along with major mechanotransductive signaling pathways and their cross-regulatory networks. Abbreviations: ECM, extracellular matrix; FAK, focal adhesion kinase; Piezo1/2, Piezo type mechanosensitive ion channels 1/2; TRPV4, Transient Receptor Potential Vanilloid 4; WNT, Wingless-related Integration Site; RhoA, Ras homolog family member A; ROCK, Rho-associated coiled-coil containing protein kinase; PI3K, Phosphoinositide 3-kinase; AKT, Protein kinase B; ERK 1/2, Extracellular signal–regulated kinase 1/2; YAP/TAZ, Yes-associated protein/Transcriptional co-activator with PDZ-binding motif. Figure created with www.BioRender.com (licensed).

Cells sense mechanical cues through membrane-localized mechanoreceptors, converting physical forces into biochemical signals that regulate proliferation, differentiation, migration, apoptosis, and ECM remodeling, thus maintaining tissue homeostasis and enabling mechanotherapy-mediated tissue repair (Figure 2).

### 3.1. Integrin–FAK Signaling Axis

Integrins are transmembrane adhesion receptors linking the ECM to the actin cytoskeleton and functioning as key mechanosensors. They detect matrix stiffness, stretch, and compression, converting these cues into biochemical signals that regulate cell fate, including proliferation, survival, and lineage specification [21,22]. Mechanical stretch or increased substrate stiffness promotes integrin clustering at the cell–matrix interface, inducing focal adhesion formation and maturation. These adhesions activate focal adhesion kinase (FAK) through autophosphorylation at Tyr397 and recruit Src family kinases, forming FAK–Src complexes that serve as central mechanotransduction hubs. Activated FAK–Src signaling modulates cytoskeletal tension, cell migration, and downstream MAPK/ERK and PI3K/Akt pathways, thereby orchestrating proliferation and differentiation [23,24,25,26].

FAK-mediated activation of RhoA and ROCK enhances actin stress fiber assembly and cytoskeletal tension, establishing the canonical integrin–FAK–RhoA/ROCK axis [27,28,29]. In mesenchymal stem cells (MSCs), mechanical activation of this axis promotes osteogenic differentiation by facilitating MAPK/ERK-dependent Runx2 expression and bone matrix deposition, while pharmacological inhibition of FAK or ROCK reduces mechanotransduction-induced osteogenesis [27,29,30]. Beyond osteogenesis, this axis also regulates MSC tenogenic differentiation under cyclic stretch or on aligned fibrous scaffolds, highlighting its relevance to tendon repair and mechanotherapy strategies [28,31].

### 3.2. Mechanosensitive Calcium Channels

Mechanosensitive calcium channels, particularly Piezo1, Piezo2, and TRPV4, constitute a major class of membrane-localized mechanoreceptors widely expressed in osteoblasts, chondrocytes, myoblasts, and mesenchymal stem cells. These channels detect diverse mechanical inputs, including matrix stiffness, stretch, compression, osmotic stress, and shear forces, and play central roles in musculoskeletal mechanotransduction [32,33,34,35]. Upon activation, mechanosensitive Ca^2+^ channels induce intracellular calcium influx, initiating downstream signaling cascades that regulate cell proliferation, differentiation, cytoskeletal organization, and extracellular matrix (ECM) remodeling, thereby contributing to tissue homeostasis and adaptive regeneration [36,37,38].

Piezo channels are rapidly activated by membrane tension and mechanical deformation, eliciting transient, high-amplitude Ca^2+^ influx that enables cells to respond promptly to acute mechanical loading. In bone, Piezo1-mediated Ca^2+^ signaling suppresses Sost expression in osteocytes via activation of the PI3K/Akt pathway, thereby directly linking mechanical sensing to osteogenic gene regulation and bone formation [39]. Piezo-dependent calcium transients further engage downstream effectors, including CaM/CaMKII, MAPK/ERK, and FAK/Src signaling, coupling membrane-level mechanosensing to focal adhesion dynamics and cytoskeletal remodeling [37,40]. In skeletal muscle, Piezo channels contribute to myofiber growth, repair, and adaptive remodeling, underscoring their importance in muscle mechanobiology and regeneration [38,41,42].

TRPV4 complements Piezo-mediated mechanotransduction by sensing moderate mechanical cues, such as osmotic swelling, hydrostatic pressure, and shear stress. TRPV4 activation typically induces slower and more sustained Ca^2+^ signaling patterns that modulate cytoskeletal organization, focal adhesion turnover, and chondrocyte homeostasis, particularly under physiological loading conditions [33,34]. Compared with the rapid and high-amplitude Ca^2+^ spikes elicited by Piezo activation, TRPV4-mediated calcium oscillations are generally lower in amplitude and more temporally stable, enabling cells to adapt to continuous or cyclic mechanical stimuli [34].

Recent transcriptomic analyses in chondrocytes have begun to elucidate how these distinct calcium temporal signatures are translated into differential gene-expression programs. Comparative studies demonstrate that activation of Piezo1 and TRPV4 drives partially non-overlapping transcriptional responses, with each channel regulating distinct gene clusters associated with cartilage homeostasis, extracellular matrix metabolism, and inflammatory signaling [34,43]. Importantly, these findings indicate that channel-specific calcium dynamics are decoded into unique transcriptional outputs, rather than converging on a uniform mechanosensitive gene program. While the precise molecular mechanisms linking calcium temporal patterns to specific transcriptional regulators remain incompletely defined, emerging evidence supports a model in which Piezo1- and TRPV4-dependent Ca^2+^ signaling differentially shapes mechanotransductive gene regulation in a context- and cell-type-dependent manner.

Together, Piezo channels and TRPV4 form an integrated yet functionally specialized mechanosensitive network that enables musculoskeletal cells to decode a broad spectrum of mechanical stimuli and translate them into precise biochemical and transcriptional responses. Their complementary roles provide important therapeutic opportunities for bone and cartilage diseases, tendon repair, and the development of mechanoresponsive biomaterials [33,34,41,42].

### 3.3. YAP/TAZ-Mediated Mechanotranscription

YAP and TAZ, nuclear effectors of the Hippo pathway, act as mechanosensitive transcriptional regulators whose subcellular localization and activity are dictated by ECM stiffness, cytoskeletal tension, and cell geometry. Under stiff or tension-rich conditions, YAP/TAZ translocate into the nucleus, partnering with TEAD transcription factors to activate genes involved in proliferation, metabolism, and differentiation [42,44,45].

Mechanically induced YAP/TAZ activation is closely coordinated with integrin–FAK–RhoA/ROCK signaling. Mechanical cues transmitted through integrins remodel the cytoskeleton, promote focal adhesion maturation, and facilitate nuclear import of YAP/TAZ [45,46]. In three-dimensional ECM or scaffold environments, mechanical signals enhance RhoA activation and cytoskeletal reorganization, enabling YAP nuclear translocation and cooperative Wnt/β-catenin signaling to support progenitor expansion [47]. Scaffold stiffness, matrix topography, and adhesion strength modulate YAP/TAZ activity and downstream transcriptional programs, which govern metabolism, lineage specification, and tissue regeneration. These processes provide a mechanistic rationale for tissue engineering and mechanotherapy strategies aimed at controlled modulation of cellular behavior [48,49].

Furthermore, nuclear mechanotransduction, including force transmission via the linker of nucleoskeleton and cytoskeleton (LINC) complex and nuclear pore dynamics, contributes to YAP/TAZ responsiveness, highlighting how external mechanical inputs can be harnessed in precision mechanotherapy to influence nuclear signaling and gene expression [44,50].

### 3.4. Cytoskeleton–Nucleoskeleton Coupling

Mechanical forces applied at the cell surface propagate through the cytoskeleton to the nucleus via lamin A/C and the LINC complex, allowing direct modulation of nuclear architecture, chromatin organization, and gene expression. Such force transmission can induce chromatin condensation, histone modifications, and nuclear stiffening, establishing a mechanical memory that shapes stem cell fate and lineage potential [51,52,53,54].

Sustained exposure to stiff microenvironments induces persistent chromatin remodeling and stable transcriptional changes, reinforcing the concept that mechanical cues can imprint durable epigenetic states [51,54]. These nuclear-level responses critically influence stem cell differentiation, proliferation, and functional output, thereby providing mechanistic support for mechanotherapy strategies that aim to guide tissue regeneration through controlled mechanical stimulation [22,50,55,56,57].

However, aging or chronic mechanical stress can compromise nuclear mechanics and LINC-mediated cytoskeleton–nucleoskeleton coupling, attenuating YAP/TAZ activity and downstream metabolic programs. This impairment diminishes regenerative capacity and cellular function, underscoring the need for personalized mechanotherapy interventions to restore or optimize mechanical signaling for effective tissue repair [52,58,59,60].

## 4. Recent Advances in Mechanotherapy for Musculoskeletal Tissue Repair and Regeneration

Mechanotherapy, defined as the application of controlled mechanical stimuli to promote tissue repair and regeneration, leverages core mechanotransduction pathways including integrin–FAK–RhoA/ROCK signaling, mechanosensitive ion channels such as Piezo, and transcriptional co-activators YAP/TAZ. These interconnected networks translate mechanical cues into cellular responses that regulate proliferation, differentiation, and extracellular matrix (ECM) remodeling. Recent advances in mechanotherapy have been reported across bone, cartilage, skeletal muscle, and tendon, highlighting its potential as a mechanism-informed and exercise-mimetic therapeutic strategy, although challenges remain in optimizing loading regimens and personalizing interventions.

### 4.1. Bone

Mechanical loading regulates bone formation, resorption, and ECM remodeling through integrated mechanotransduction networks. Integrin–FAK signaling and downstream RhoA/ROCK-mediated cytoskeletal tension enable osteoblasts to sense matrix stiffness and mechanical stress, thereby controlling cell adhesion, osteogenic differentiation, and osteoclast-related activities [61,62]. Wnt/β-catenin signaling acts as a central transcriptional mediator, integrating cytoskeletal inputs to regulate osteogenic gene expression, RANKL/OPG balance, and ECM organization [61,63].

Low-intensity pulsed ultrasound (LIPUS) has emerged as a noninvasive mechanotherapeutic stimulus. LIPUS reorganizes integrin-based focal adhesions, activates vinculin and FAK signaling, and stimulates small GTPases such as Rac1, enhancing cytoskeletal dynamics and cellular mechanosensitivity [64]. LIPUS also modulates osteoblast–osteoclast crosstalk, activates Piezo1-mediated Ca^2+^ influx, and engages downstream YAP/Lamin A/C signaling, collectively promoting osteogenic differentiation and bone matrix deposition [65,66,67,68,69]. Furthermore, ultrasonic stimulation stabilizes β-catenin via GSK3β inhibition, facilitating Wnt-mediated transcription and nuclear translocation [70].

Although a single unified experimental framework connecting integrin–FAK, Piezo1, YAP/TAZ, and Wnt/β-catenin signaling is still lacking, cumulative evidence supports a coordinated mechanotransduction network through which LIPUS and mechanical loading enhance bone formation, ECM remodeling, and peri-implant osseointegration. These mechanistic insights provide a foundation for precision bone mechanotherapy based on defined mechanical inputs and tissue-specific responses.

### 4.2. Cartilage

Physiological mechanical loading is critical for cartilage homeostasis and for maintaining the chondrocyte phenotype. Cyclic compressive and shear forces within physiological ranges upregulate type II collagen and proteoglycan synthesis while suppressing matrix-degrading enzymes, including matrix metalloproteinases (MMPs) and ADAMTS, thereby preserving ECM integrity [71,72,73]. Conversely, excessive mechanical stress can activate YAP/TAZ signaling and suppress chondrogenic gene expression, underscoring the dose-dependent nature of mechanical regulation [71].

Anabolic mechanotransduction in cartilage is primarily mediated by mechanosensitive calcium channels such as TRPV4, coordinated with integrin–FAK-dependent adhesion signaling. Downstream YAP/TAZ and Wnt/β-catenin pathways integrate mechanical inputs to regulate ECM synthesis and remodeling [71,73,74,75]. While each component of this cascade has been individually validated, a fully integrated mechanotransduction loop in chondrocytes remains to be elucidated.

Future studies integrating live-cell imaging of mechanosensitive Ca^2+^ dynamics with single-cell or spatial multi-omics under defined mechanical loading conditions may help bridge this gap and establish a spatiotemporally resolved mechanotransduction framework linking force sensing to transcriptional outputs.

Noninvasive mechanical interventions, particularly LIPUS, demonstrate regenerative potential in cartilage repair. LIPUS enhances MSC chondrogenic differentiation, including umbilical cord-derived MSCs, by upregulating Sox9, type II collagen, and aggrecan while suppressing inflammatory mediators such as TNF-α [76]. In vivo studies show that LIPUS combined with MSC transplantation improves ECM deposition and repair quality. Additionally, LIPUS promotes MSC migration and autophagy-mediated exosome release, supporting chondrocyte proliferation and matrix synthesis in osteoarthritis and focal cartilage injury models [77,78].

Despite these advances, variability in joint biomechanics, age-related decline in mechanosensitivity, and heterogeneous injury microenvironments complicate the optimization of loading-based protocols, emphasizing the need for personalized cartilage mechanotherapy guided by biomechanical and cellular feedback.

### 4.3. Skeletal Muscle

The mechanobiology of skeletal muscle regeneration has been increasingly elucidated. Satellite cells express the mechanosensitive ion channel Piezo1, and loss of Piezo1 severely impairs regenerative capacity. Mechanical activation of Piezo1-mediated Ca^2+^ signaling induces intracellular Ca^2+^ elevations that regulate satellite cell proliferation and differentiation programs [79]. Piezo1 signaling also suppresses p53-dependent senescence, preserving stem cell regenerative potential and positioning mechanical loading as an exercise-mimetic, anti-aging mechanism that maintains muscle regenerative capacity across the lifespan [80].

Integrin–FAK signaling provides essential adhesion-mediated cues for satellite cell plasticity. Age-related reductions in fibronectin weaken integrin engagement and impair regeneration [81], whereas moderate mechanical loading restores integrin–FAK signaling and supports downstream YAP/TAZ activation [82]. Conversely, excessive ECM stiffening following injury can sustain YAP/TAZ nuclear localization, prolong satellite cell activation, and delay differentiation [83].

Furthermore, the structural organization and function of the myotendinous junction (MTJ) and the costamere are critical for skeletal muscle mechanotransduction. The MTJ connects muscle fibers to tendons, transmitting contractile forces to the extracellular matrix and providing a focal point for integrin clustering and FAK activation. The costamere links the sarcolemma to the Z-disk, coordinating cytoskeletal tension and enabling force distribution across the muscle fiber. Both structures act as subcellular mechanosensitive hubs that integrate mechanical inputs with Piezo1-mediated Ca^2+^ influx, integrin–FAK–YAP/TAZ signaling, and Wnt/β-catenin pathways, ultimately regulating satellite cell behavior, myofiber alignment, and muscle regeneration [79,81,82].

Wnt/β-catenin signaling further governs myogenic differentiation and hypertrophic adaptation by promoting myogenic commitment [84,85] and modulating the follistatin–myostatin and activin axis to improve muscle tissue quality [82]. Thus, mechanical cues function not merely as activation signals but as context-dependent regulators of muscle regeneration, aging, and structural adaptation.

### 4.4. Tendon

Tendons rely on physiological mechanical loading to maintain homeostasis and tendon–bone interface integrity. Tensile, shear, and compressive stresses activate integrin–FAK signaling together with ILK and mTOR pathways, promoting ECM remodeling, collagen synthesis, and mechanical strength [79,80,81]. β1 integrin and ILK activation enhance type I collagen and matrix protein expression, whereas mTOR inhibition suppresses remodeling, highlighting the dosage sensitivity of tendon mechanotherapy [80].

The transcription factor scleraxis (SCX) functions as a mechano-inducible regulator of tendon ECM. Mechanical loading upregulates SCX, directing tendon-specific matrix deposition and structural organization [81,82]. Conversely, stress deprivation or insufficient mechanical stimulation compromises cell adhesion, weakens ECM integrity, and induces pro-inflammatory phenotypes, emphasizing the importance of precisely controlled mechanical environments in tendon repair strategies [83,84].

LIPUS has demonstrated translational potential by accelerating tendon–bone interface healing through modulation of macrophage polarization and improvement of structural integration [85]. Preclinical studies confirm that LIPUS enhances ECM deposition, biomechanical restoration, and functional recovery in tendon, ligament, and soft tissue interface injury models, supporting its use as a noninvasive mechanotherapeutic modality [86]. Parallel efforts to develop ECM-functionalized tendon substitutes with tunable mechanical properties aim to recapitulate native tendon architecture and function, further extending scaffold-guided mechanotherapy toward clinical translation [87].

## 5. Engineering Innovations in Mechanotherapy

**Figure 3:**
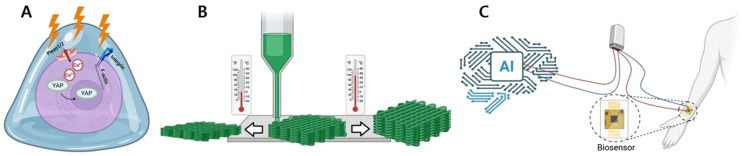
Schematic of engineering innovations in mechanotherapy, showing (**A**) mechanosensitive biomaterials, (**B**) 4D printed shape-morphing scaffolds, and (**C**) artificial intelligence-enabled wearable mechanotherapy systems. Figure created with www.BioRender.com (licensed).

The translation of mechanotherapy into precision clinical interventions has been accelerated by engineering innovations in three major domains: mechanosensitive biomaterials, 4D printed shape-morphing scaffolds, and artificial intelligence (AI) assisted wearable mechanical stimulation systems (Figure 3). These approaches extend the capacity of mechanotherapy beyond traditional exercise or device-based loading, enabling programmable, responsive, and personalized modulation of tissue regeneration.

### 5.1. Advances in Mechanosensitive Biomaterials

Mechanosensitive biomaterials have transformed mechanotherapy by providing engineered platforms that not only transmit mechanical cues but also actively sense and transduce them into biochemical and biophysical signals, thereby enabling precise control over cellular mechanobiological responses [60,88]. These systems are conceptually inspired by the mechanotransductive principles underlying exercise-based rehabilitation and aim to recapitulate the dynamic mechanical environments experienced by tissues in vivo [89]. Accordingly, biomaterials are increasingly designed to mimic the viscoelastic, time-dependent properties of native extracellular matrices (ECM), exposing resident or transplanted cells to physiologically relevant mechanical inputs that more closely reflect in vivo loading conditions [90].

Hydrogels with dynamic and reversible crosslinking networks—established through covalent bonds, host–guest interactions, or ion-mediated associations—exhibit adaptive mechanical behavior under external loading, thereby reproducing key features of tissue viscoelasticity such as stress relaxation and strain-rate sensitivity [91,92]. These mechanically responsive properties critically regulate intracellular tension, cytoskeletal organization, and mechanosensitive signaling, ultimately influencing ECM synthesis and phenotype maintenance in load-bearing tissues such as cartilage [93,94]. In particular, tunable alginate-based hydrogels with adjustable crosslinking density and relaxation kinetics have been shown to enhance chondrocyte mechanosensing and matrix deposition, highlighting the importance of temporal mechanical cues in regenerative outcomes [95].

Piezoelectric polymers, including polyvinylidene fluoride (PVDF) and its copolymers with trifluoroethylene, represent a distinct class of mechanosensitive biomaterials capable of directly converting mechanical deformation into localized electrical potentials [96,97,98,99]. These electrically active scaffolds modulate cell adhesion, Ca^2+^ dynamics, and ECM production, thereby complementing classical mechanotransduction pathways mediated by integrins, mechanosensitive ion channels, and YAP/TAZ signaling. Importantly, this intrinsic electromechanical coupling enables the generation of bioelectrical signals directly from endogenous mechanical activities, such as muscle contraction, joint motion, or physiological loading, without the need for external power sources. Such self-powered electrical stimulation is particularly attractive for deep tissues, where conventional electrical therapies are limited by electrode placement, wiring, and energy delivery constraints. Consistent with this concept, recent review studies have emphasized that piezoelectric biomaterials can provide endogenous electrical stimulation driven by mechanical loading alone and exhibit promising translational potential across multiple tissue engineering applications, including soft tissue, bone, and nerve regeneration [100,101,102]. By locally translating mechanical cues into electrical signals in situ, piezoelectric scaffolds offer a unique strategy to integrate mechanical and electrical stimulation while minimizing device complexity and invasiveness.

Beyond bulk material properties, biochemical functionalization further enhances mechanosensitivity at the cell–material interface. Scaffolds modified with integrin-binding peptides, such as RGD and IKVAV, promote focal adhesion assembly, cytoskeletal tension, and downstream mechanosignaling, thereby amplifying cellular responsiveness to applied or endogenous mechanical cues [103,104,105]. When integrated into hydrogels or self-assembled nanofiber systems, these approaches enable controlled modulation of cell proliferation, migration, and ECM deposition. Future advances are expected to combine micro- and nano-topographical patterning with tunable macroscopic mechanical compliance, allowing precise regulation of local stress distributions and cell alignment. Such strategies align with emerging concepts of mechanome-guided regenerative rehabilitation and YAP/TAZ-mediated mechanotransduction, further expanding the therapeutic scope of biomaterial-assisted mechanotherapy [8,42].

### 5.2. 4D Printed Shape Morphing Scaffolds

4D printed scaffolds extend conventional 3D constructs by incorporating stimuli-responsive materials that change shape or mechanical properties over time or in response to environmental cues such as temperature, pH, humidity, or electromagnetic fields [106,107,108,109,110]. These dynamic scaffolds autonomously adapt their geometry during tissue growth and deliver programmed mechanical stimuli, including cyclic stretching, localized compression, or microscale deformation, providing precise mechanical cues for guided regeneration [106,107,108,109,111].

Recent developments in 4D printing have produced hydrogels and shape-morphing scaffolds capable of dynamically regulating morphology and stiffness, thereby creating tunable microenvironments that promote cell migration, alignment, and tissue remodeling [107,108,109,111,112,113,114,115]. Platforms such as muscle-mimetic thermomechanically adaptive hydrogels, printable alginate–methylcellulose composites, 3D printed collagen scaffolds, and bio-piezoelectric smart scaffolds have demonstrated compatibility with dynamic mechanical conditions in cardiac, skeletal muscle, and bone tissue engineering [108,109,112,116]. These constructs provide innovative physical platforms to deliver programmable mechanotherapy with tissue-specific precision.

### 5.3. Artificial Intelligence Assisted Wearable Mechanotherapy Systems

Advances in flexible sensors, wireless power delivery, and AI-driven algorithms are shifting mechanotherapy from empirical, open-loop protocols to real-time, closed-loop adaptive control. Flexible tactile sensors continuously monitor tissue stress and movement patterns, providing immediate feedback for personalized mechanical stimulation [117,118]. Recent progress in wireless powering strategies, including the use of metamaterials for efficient energy transfer to implantable medical devices, enables long-term, stable monitoring and modulation of in vivo mechanical cues [119]. Wearable devices and assistive robotic systems increasingly quantify forces and deliver reproducible mechanical inputs, enhancing standardization and efficacy in clinical rehabilitation [120,121,122,123].

At the computational level, AI and machine learning optimize loading parameters by analyzing electromyography, movement patterns, and multimodal sensor data to autonomously adjust intensity, frequency, and duration of mechanical stimulation [124,125]. These closed-loop systems integrate real-time feedback to support individualized mechanotherapy, moving beyond static, empirically defined protocols [118,126]. By combining responsive biomaterials, programmable scaffolds, and AI-assisted wearable platforms, modern mechanotherapy achieves precision, personalization, and dynamic adaptability in tissue repair and regeneration.

## 6. Precision Mechanotherapy: Clinical Translation and Future Directions

Mechanotherapy has emerged as a clinically relevant approach in regenerative rehabilitation, aiming to enhance tissue repair and functional recovery through the controlled application of mechanical stimuli to injured or degenerative tissues [8]. Evidence from preclinical and clinical studies demonstrates that regulated loading modalities, including whole-body vibration, targeted resistance training, and localized mechanical stimulation, can promote musculoskeletal regeneration by increasing bone density, improving tendon remodeling, and restoring neuromuscular function [89,127,128]. Mechanical inputs can also modulate articular cartilage matrix turnover, attenuate degenerative progression, and support cartilage repair [129]. From a mechanobiological perspective, the intrinsic mechanical properties of biomaterials act as regulatory cues, directing cell behavior independently of biochemical signals [130]. In bone, interstitial fluid shear stress, cyclic compression, and strain gradients orchestrate matrix remodeling, balance osteoblast and osteoclast activity, and regulate lineage specification, collectively driving tissue deposition, mineralization, and structural adaptation [128].

Despite these promising outcomes, clinical responses to mechanotherapy remain heterogeneous, underscoring the need for patient stratification based on mechanical phenotypes [44,45,131]. Variations in tissue stiffness, cellular mechanosensitivity, and age-related impairments in nuclear force transmission significantly influence therapeutic efficacy [44,45,131]. Mechanistic studies indicate that YAP/TAZ signaling, nuclear lamins, and related mechanosensitive regulators function as force-responsive transcriptional switches that govern cell fate determination and phenotypic transitions [44,45,131]. In chondrocytes and mesenchymal cells, mechanical stimulation can alter chromatin accessibility, influence mechanical memory, and modulate cellular plasticity [55,59,131]. These insights highlight that mechanotherapy should be administered with the precision of a pharmacological regimen, requiring quantitative control over loading magnitude, frequency, waveform, and duration, along with iterative adjustments based on cellular and tissue-level feedback [55,59,131].

Digital health technologies are accelerating the transition toward personalized and adaptive mechanotherapy. Wearable biomechanical sensors, machine learning-based rehabilitation algorithms, and robotic assistive devices enable real-time monitoring of gait, joint kinematics, and muscle activity, supporting individualized and data-driven mechanical interventions [121,123]. Artificial intelligence and machine learning integrated with wearable sensors have been shown to enable real-time assessment, outcome prediction, and personalized adaptations of rehabilitation programs, thereby enhancing patient-specific care and therapeutic precision in clinical settings [132]. These technologies allow continuous acquisition and interpretation of complex sensor data, providing actionable feedback that can be used to adjust mechanical dosing and optimize therapeutic progression [133]. Such adaptive systems extend mechanotherapy beyond static prescriptions toward dynamic, closed-loop rehabilitation guided by individual responses and recovery trajectories [134].

Mechanosensitive biomaterials further enhance this precision, with programmable scaffolds, including 4D-printed constructs capable of predictable dynamic deformation, delivering targeted mechanobiological cues for cartilage repair [111]. High-fidelity ECM-mimetic scaffolds, such as pure collagen bioprinted constructs, recreate controlled microenvironments that closely emulate native matrix and support essential cell–matrix interactions in load-bearing tissues [115].

Successful clinical translation of mechanotherapy requires a robust understanding of mechanobiology alongside rigorous safety and regulatory frameworks. Digital twin technologies enable patient-specific biomechanical modeling and individualized treatment planning [135], facilitating integrated assessment of device–material–algorithm–patient systems beyond single-device evaluation [136,137]. Mechanically responsive 4D-printed scaffolds have demonstrated controllable deformation, tunable mechanical properties, and favorable biocompatibility in both in vitro and in vivo settings [138,139,140]. Ensuring clinical viability demands evaluation of reproducibility, long-term functional stability, and clearly defined safety thresholds for mechanoresponsive materials and mechanical dosing regimens [141,142]. As digital and AI-based technologies become increasingly integrated into rehabilitation platforms, mechanotherapy is advancing toward precision, standardization, and prescription-based management. This evolution requires careful consideration of ethical frameworks, algorithmic transparency, and model interpretability to ensure safety, accountability, and traceability in clinical decision-making [143,144].

## 7. Conclusions

Mechanotherapy is transforming regenerative rehabilitation by shifting from empirical loading approaches toward precise, mechanism-based interventions guided by cellular mechanotransduction. Accumulating evidence demonstrates that mechanical stimuli inherent to exercise and physical therapy exert their therapeutic effects through conserved molecular pathways that translate physical forces into gene regulatory programs. Recent advances in mechanosensitive biomaterials, 4D-printed shape-morphing scaffolds, and artificial intelligence-enabled wearable systems now allow mechanical stimulation to be quantifiable, programmable, and tailored to patient-specific mechanical phenotypes.

By integrating mechanobiological insights with engineering and digital innovations, mechanotherapy is progressing toward standardized, prescription-based mechanical interventions, in which loading parameters can be defined, monitored, and adapted with clinical rigor. Conceptually, mechanotransduction can be viewed as a biological translation system, whereby the physical language of movement—such as stretching, compression, or shear—is decoded by cellular mechanosensors into biochemical and transcriptional instructions that direct tissue maintenance and regeneration. The continued clinical translation of mechanotherapy will therefore require cross-disciplinary training that bridges biology, engineering, and rehabilitation sciences, enabling the next generation of clinicians and therapists to implement safe, mechanism-informed, and personalized mechanical prescriptions.

## Data Availability

No new data were created or analyzed in this study. Data sharing is not applicable to this article.
